# cMyc/miR-125b-5p Signalling Determines Sensitivity to Bortezomib in Preclinical Model of Cutaneous T-Cell Lymphomas

**DOI:** 10.1371/journal.pone.0059390

**Published:** 2013-03-19

**Authors:** Valentina Manfè, Edyta Biskup, Ayalah Willumsgaard, Anne Guldhammer Skov, Dario Palmieri, Pierluigi Gasparini, Alessandro Laganá, Anders Woetmann, Niels Ødum, Carlo Maria Croce, Robert Gniadecki

**Affiliations:** 1 Department of Dermatology, Bispebjerg Hospital, Copenhagen, Denmark; 2 Institute of Medical Microbiology and Immunology, University of Copenhagen, Copenhagen, Denmark; 3 Department of Molecular Virology, Immunology, and Medical Genetics, Comprehensive Cancer Centre, Ohio State University, Columbus, Ohio, United States of America; 4 Faculty of Health Sciences, University of Copenhagen, Copenhagen, Denmark; Karolinska Institutet, Sweden

## Abstract

Successful/effective cancer therapy in low grade lymphoma is often hampered by cell resistance to anti-neoplastic agents. The crucial mechanisms responsible for this phenomenon are poorly understood. Overcoming resistance of tumor cells to anticancer agents, such as proteasome inhibitors, could improve their clinical efficacy. Using cutaneous T-cell lymphoma (CTCL) as a model of the chemotherapy-resistant peripheral lymphoid malignancy, we demonstrated that resistance to proteasome inhibition involved a signaling between the oncogene cMyc and miR-125b-5p. Bortezomib repressed cMyc and simultaneously induced miR-125b-5p that exerted a cytoprotective effect through the downmodulation of MAD4. Overexpression of cMyc repressed miR-125b-5p transcription and sensitized lymphoma cells to bortezomib. The central role of miR-125b-5p was further confirmed in a mouse model of T-cell lymphoma, where xenotransplantation of human CTCL cells overexpressing miR-125b-5p resulted in enhanced tumor growth and a shorter median survival. Our findings describe a novel mechanism through which miR-125b-5p not only regulates tumor growth *in vivo,* but also increases cellular resistance to proteasome inhibitors *via* modulation of MAD4.

## Introduction

Low grade lymphomas are a group of haematological malignancies characterised by a slow rate of proliferation and frequent relapse after traditional chemotherapy [1]. Proteasome inhibitors are intensively studied for the therapy of these diseases, since these drugs target mitotically quiescent cells. Bortezomib (Velcade®), the most frequently used proteasome inhibitor, has shown to be promising for a range of refractory lymphomas including relapsed mantle cell lymphoma, peripheral T-cell lymphoma and cutaneous T-cell lymphoma [2]. However, the overall response rates are in the range of 30–50% indicating that more than half of the patients do not benefit from the treatment due to tumor cell resistance [3]–[5]. Delineation of the mechanisms involved in the resistance to proteasome inhibitors would lead to new, improved treatment strategies [6]. It has already been reported that functional mutation in proteasome components or activation of alternative mechanisms of protein degradation (*e.g.* aggresome pathway) may bypass the effects of the inhibition of the proteasome signaling [6].

Cutaneous T-cell lymphomas (CTCLs) comprising the two major clinical entities, mycosis fungoides and Sézary syndrome, are the most common extranodal T-cell lymphomas and constitute typical examples of recalcitrant, chemotherapy resistant and low- to medium grade lymphoid malignancies [7], [8]. Proteasome inhibitors show activity in CTCL, but the rate of complete remissions is low [9]–[11]. Taking advantage of the recent discovery that inhibition of cMyc leads to drug resistance in melanoma and myeloma [12]–[14], we aimed to investigate whether cMyc is also involved in the regulation of the resistance to bortezomib in CTCL. cMyc is an attractive target in CTCL since increased expression is observed in the majority of Sézary syndrome patients and in a high proportion of advanced mycosis fungoides [15], [16]. MicroRNAs (miRNAs) are an abundant class of small, non-coding endogenous RNAs (18–25 nucleotides), that target mRNAs causing post-transcriptional inhibition of protein expressions [17]. We were particularly interested in the possible involvement of the miRNA regulatory circuits due to the well documented role of miRNAs in the regulation of cellular signaling and cancer development [18], [19].

We describe here a novel regulatory circuit where miR-125b-5p directly targets MAD4 and modulates cMyc accumulation. We document the importance of this pathway both for tumor growth *in vivo* and for the resistance of CTCL cells to chemotherapy.

## Materials and Methods

### Ethic Statement

Skin biopsies were collected from 17 patients with mycosis fungoides after written informed consent. The study was approved by the Ethics Committee of Copenhagen and Frederiksberg.

The animal treatments were performed in strict accordance with the recommendations in the Guide for the Care and Use of Laboratory Animals of the National Institutes of Health [20]. All procedures were carried out with the approval of the Danish National Animal Ethics Committee (Per 2012/DY/2930/00748).

### Cell Culture and Tissue Procurement

Two CTCL cell lines have been used: MyLa2000 derived from a plaque biopsy of a patient with mycosis fungoides [21] and SeAx derived from peripheral blood of patients with Sézary syndrome [22]. Cell lines were authenticated by analysis at the Section of Forensic Genetics, University of Copenhagen. MyLa and SeAx cells were cultured in DMEM containing 4.5 g/l glucose, 10% fetal bovine serum (FBS) and at 37°C under 5% CO_2_. *In situ* hybridization (ISH) was performed on paraffin sections of 17 patients with mycosis fungoides (13 males and 4 females; mean age 69 years; range 48 to 92 years; 11 plaque/patch (T2) and 6 tumour (T3) stadium). Patient diagnoses were confirmed by an expert pathologist in accordance with the WHO-EORTC classification [8].

### siRNA and miRNA Transfection

SeAx cells were transfected as previously described [23]. In the case of MyLa cells, transfection was carried out using Amaxa machine (Lonza, Basel, Switzerland) and Nucleofector Kit-T (Cat VCA-1002). 100 nM miRIDIAN miRNA Mimics (Thermo Scientific, Chicago, Il) and miRCURY™ LNA Inhibitor (Exiqon, Vedbaek, Denmark) were used for specific overexpression and inhibition of miR-125b-5p, respectively. Small Interfering RNA was used at 50 nM for the specific knockdown of MAD4 and cMyc (Ambion Inc., Austin, TX, USA).

### Bioinformatics Analysis

The prediction of binding sites for cMyc in the region 10 kb upstream of pre-miR-125b-1 and pre-miR-125b-2 was performed by TESS-Transcription Element Search System [24].

The prediction of targets for miR-125b-5p was performed using the online tools Targetscan [25] and PicTar [26].

### Plasmids and Luciferase Reporter Vector

The expression plasmids for cMyc and the corresponding empty vector (PCDNA3.1, Invitrogen, San Diego, CA) were kindly provided by Dr. Carlo M. Croce (Department of Molecular Virology, Immunology, and Medical Genetics, Comprehensive Cancer Centre, Ohio State University, Columbus).

The 3′ UTR of the human MAD4 gene was PCR-amplified using the primers:

MAD4-FW 5′-GGG CTT CTC ATG AGC- 3′.

MAD4-REV 5′-GGG CCT GGC TGC ATC G-3′.

The PCR products were digested using XHO and NOT and cloned downstream of the multi cloning site of psiCheck2 vector (Promega, Madison, WI).

To delete the seed sequence of miR-125b-5p on the *MXD4* 3′UTR, we used the Quick-Change Site-Directed Mutagenesis kit (Agilent Technologies, Santa Clara, CA) and the primers:

MUT-MAD4-FW 5′-CCA CCC AGC GTC CCT GTC CCT CCG-3′.

MUT-MAD4-RV 5′-CGG AGG GAC AGG GAC GCT GGG TGG-3′.

For the reporter luciferase assay Hek-293 were cotransfected in a 12 well-plate with 1 µg psiCheck2-MAD4 and/or psiCheck2-Mut and 100 nM of miR-125b-5p or scrambled sequence miRNA control (Thermo Scientific). Dual Luciferase Assay (Promega) was performed 24h after transfection according to the manufacturer’s instructions.

The promoter assay was performed by amplification of the DNA fragments containing the cMyc putative regulatory region upstream to pre-miR-125b-5p (BS2) and successively cloning in pGL3basic (Promega). Hek-293 were cotransfected in a 12 well-plate with 2 µg of pGL3basic empty vector or of pGL3basic containing the above genomic fragments, 200 ng Renilla luciferase expression construct pRL-TK (Promega) and 2 µg of cMyc overexpressing plasmid.

Cells were harvested after 48 h and assayed with Dual Luciferase Assay (Promega) according to the manufacturer’s instructions. The primers used for the cloning were the following:

BS2-FRW 5′-TTC CAG CCC CTC CCT CCA C-3′.

BS2-REV 5′-CTG AAG AGA AAA TAG GAG GCA TA-3′.

### Gene Expression Analysis

Total RNA (TRNA) was isolated from cells by the QIAcube apparatus (Qiagen, Hilden, Germany), using RNeasy MiniKit (Qiagen). RNA concentration and purity were assessed using NanoDrop ND-1000 (Thermo Scientific). RNA was transcribed into cDNA (1 µg TRNA per reaction), using universal primers (Promega), according to the manufactures instructions. qRT-PCR reaction was carried out with 1∶10 diluted cDNA template, unlabeled primers specific for genes of interest and FAM labeled probes, using the Applied Biosystems kit. The reaction conditions were: 10 min activation step (95°C), followed by 40 repeated cycles (15 s–95°C and 60 s–60°C). Fluorescence data were captured and analyzed by ABI Prism 7000 SDS software (Applied Biosystems). Fold-change in genes of interest expression was normalized for GAPDH expression (Applied Biosystems).

### Human miRNA PCR Array and miRNA Expression Analysis

miRNA enriched RNA fraction was isolated from cells by the QIAcube apparatus (Qiagen), using miRNeasy Mini kit (Qiagen). RNA concentration and purity were assessed using NanoDrop ND-1000 (Thermo Scientific). Analysis of changes in the miRNome profile, induced by cMyc silencing, was performed using Human miRNome miScript miRNA PCR Array MIHS-216Z (Qiagen). Briefly, 150 ng of miRNA, isolated from MyLa cells transfected either with cMyc siRNA or scambled RNA was transcribed into cDNA, using miScript II RT Kit (Qiagen) and expression patterns of 1008 human miRNAs were analyzed by qRT-PCR, according to manufacturer’s protocol. Data analysis was performed using the miScript miRNA PCR Array Data Analysis Web Portal (http://pcrdataanalysis.sabiosciences.com/mirna/arrayanalysis.php) using ≥4 fold-regulation cutoff. A panel of snoRNA/snRNA (SNORD61, SNORD68, SNORD72, SNORD95, SNORD96A, RNU6-2) was used to normalize array data.

For the miRNA expression analysis, RNA was transcribed into cDNA using High Capacity cDNA Reverse Transcription Kit (Applied Biosystems, Foster City, CA), with primers specific for miRNA of interest (Applied Biosystems). Subsequently, the cDNA template was diluted 1∶15 and the quantitative RT-PCR reaction was carried out as described above for gene expression analysis. miR-191 was used as a reference for the normalization of RT-PCR data (Applied Biosystems).

### Chromatin Immunoprecipitation (ChIP) Assay

ChIP assay was performed as previously [27] in Seax and MyLa cells using the ChIP assay kit (Upstate Biotechnology, Lake Placid, NY) and DNA-protein complexes were immunoprecipitated using anti-cMyc antibody (Santa Cruz, Heidelberg, Germany). Purified chromatin was subjected to PCR using the following primers:

BS2-FRW 5′-TTC CAG CCC CTC CCT CCA C-3′.

BS2-REV 5′-CTG AAG AGA AAA TAG GAG GCA TA-3′.

### *In situ* Hybridization (ISH) and Immunohistochemistry (IHC)

ISH was performed on paraffin sections of 17 patients with mycosis fungoides as previously described [23]. Three different mercury locked nucleic acid (LNA) miRNA Detection Probes (Exiqon, Vedbaek, Denmark) were used; hsa-miR-125b-5p (40 nM in a formamide-free ISH buffer), scrambled probe (40 nM) or U6 probe (1 nM as positive control). The same paraffin sections were also analyzed by IHC for cMyc (1∶200 in Dako antibody diluent; Thermo Scientific). Briefly, paraffin was removed using EZ-prep (Ventana Medical Systems Inc, Tuscon, AZ) and the slides were pre-treated with mild Cell Conditioning 1 buffer (CC1 pH 8.5, Ventana) for 64 min and incubated with cMyc antibody for 32 min at 36°C. The reaction was visualized using Ventana Opti View DAB-kit. Afterwards the sections were counterstained with Ventana hematoxylin.

Paraffin sections of tumor tissue from NSG mice, were analyzed by ISH using hsa-miR-125b-5p, scrambled and U6 probes.

### Apoptosis Assessment

Cells were treated with bortezomib (MG341) (Selleck, West Paterson, NJ). In order to assess the apoptotic rate, cells were double-stained with annexin V-FITC and PI according to the manufacturer’s protocol (Beckman-Coulter, Fullerton, CA) and analyzed by means of flow cytometry (Beckman-Coulter, Fullerton, CA) as described previously [23].

### Western Blot

Whole cell extracts and western blot analysis were performed as described previously [23]. The following primary antibodies were used: cMyc from Epitomics (CA; Cat.#: 1472-1, dilution 1∶500), MAD4 from Sigma Aldrich (St. Louis, MO; Cat.#: SAB2500612; dilution 1∶250) [23] and anti–β-actin (Sigma Aldrich; ab8227; dilution 1∶20.000). Immunoreactivity was detected and quantified with the infrared Odyssey imaging System (Li-Cor).

### Establishment of the Mouse Tumor Model

Establishment of human lymphoma model was performed using male NOD SCID gamma (NSG) mice. Suspensions of MyLa cells, transfected with miR-125b-5p or scrambled RNA (Thermo Scientific, Chicago, Il), were injected subcutaneously in the flanks (0.8×10^6^ cells/100 ul per flank; 6 mice in each group). Animals were weighed twice a week and the tumor size was measured three times a week by caliper. Tumor volume was calculated using the formula:
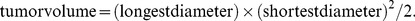
(1)


When the longest tumor diameter exceeded 12 mm, mice were sacrificed by cervical dislocation. Postmortem examination included tumor size and weight measurements. Tumor growth rate was calculated using the formula (1) and compared between the miR-125b-5p and scrambled control groups. Slopes of the linear regression lines were compared and the *P*<0.05 was considered significant.

### Statistic Analysis

Continuous data are reported as means with standard deviation (SD) and the differences were evaluated by the two-tailed Student’s *t*-test. qRT-PCR data were analyzed using the ΔΔCT method and Wilcoxon two group test was used to analyse ΔCT values. The correlation between miR-125b-5p and cMyc in mycosis fungoides specimens was calculated using the Spearman correlation coefficient. The effect of miR-125b-5p on *in vivo* tumorigenesis was assessed by Kaplan–Meier method and the survival curves were compared using Log-Rank Mantel Cox test. *P<0.05 was considered significant. Statistical analysis was performed by the SPSS Version 17.0 (SPSS Inc., Chicago, Il), GraphPad Prism Version 4.03 (GraphPad Software Inc., San Diego, CA) or Excel (Microsoft Corp., Redmond, WA).

## Results

### cMyc Represses miR-125b-5p in CTCL

**Figure 1:**
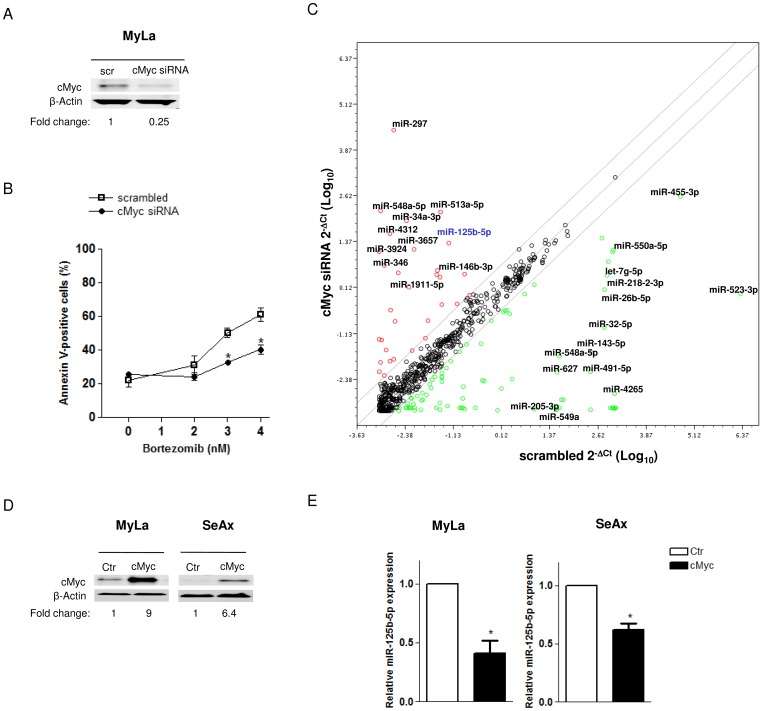
Identification of miRNAs regulated by cMyc in CTCL. (A) Efficient downregulation of cMyc protein after 24h cMyc silencing in MyLa cells. cMyc protein bands were normalized to β-actin band intensities and relative protein expression levels are reported below the corresponding Western blot bands. (B) Effect of cMyc silencing on the apoptosis induced by 48h treatment with bortezomib in MyLa cells. Apoptosis was measured by annexin V and propidium iodide staining for flow cytometry. Bar graph shows the percentage of annexin V-positive cells in the total cell population. Data are presented as mean ±SD (*P< 0.05). (C) Scatter plot of the human miRNome miScript miRNA PCR Array profile depicting the changes in miRNA expressions after cMyc silencing in MyLa cells. The graph was obtained by plotting the normalized log_10_ miRNA expression (2^−Δct^) in cMyc-knockdonwn MyLa cells (y-axis) divided by the one in the control sample (x-axis). miRNAs were sorted using a ≥4 fold-regulation cutoff and miRNAs upregulated and downregulated are showed with red and green dots, respectively. (D) Overexpression of cMyc protein in CTCL cells (MyLa and SeAx) transfected with cMyc expression plasmid (cMyc) compared to the ones transfected with the control vector (Ctr). cMyc protein levels are reported as in Figure 1A. (E) qRT-PCR showing the miR-125b-5p downregulation after cMyc accumulation. Data are presented as mean ± SD (*P< 0.05).

We have previously demonstrated that the free oxygen radical mediated proteasome blockade is a strong inducer of apoptosis in different CTCL cell lines [23], [28]. In MyLa cells, representing tumor cells of mycosis fungoides, silencing of cMyc resulted in resistance to bortezomib, analogously to what has been shown in multiple myeloma, melanoma, breast and cervical carcinomas [12]–[14] (Figure 1A, B). The involvement of cMyc was also shown in the SeAx cell line derived from Sézary syndrome, where the ectopic expression of cMyc sensitized CTCL cells to apoptosis after bortezomib (Figure S1A, B). In order to determine whether cMyc modulates miRNA expressions we examined the changes in global miRNA profile in cMyc-knockdown (cMyc-KD) MyLa cells (Figure 1C, Table S1). Using a ≥4 fold-regulation cutoff, we identified 30 upregulated and 84 downregulated miRNA species (Figure 1C). In the list of deregulated miRNAs we recognized few miRNAs previously shown to be modulated by cMyc including miR-34a-3p and miR-125b-5p [29], [30], miR-17-3p [31] and miR-20a [32], [33], let-7 family and miR-9-5p [34]. Several research studies have defined a role for miR-34a, miR-17 and let-7 families in lymphoma and other hematopoietic neoplasms [35]–[41]. We focused on miR-125b-5p since its function in T-cell lymphoma has not yet been investigated. We showed that miR-125b-5p is tightly regulated by cMyc as exemplified by its increased expression in cMyc-KD cells and its repression in the cMyc overexpressing MyLa and SeAx (Figure 1C–E).

**Figure 2:**
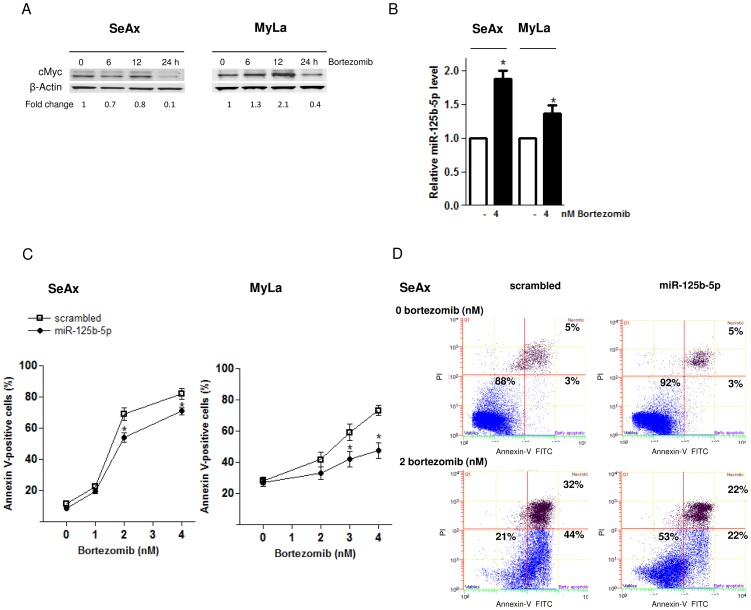
miR-125b-5p induces cell resistance to bortezomib. (A) cMyc protein levels after bortezomib treatment (4 nM) at the indicated time points in CTCL cells (SeAx and MyLa). β-actin was used as a loading control and the cMyc band were quantified as in Figure 1A. (B) qRT-PCR showing the miR-125b-5p upregulation after 24h treatment with bortezomib (4 nM). (C) Effect of miR-125b-5p overexpression on the apoptosis induced by 48 h treatment with bortezomib. Percentages of annexin V-positive cells in the total cell population is shown. (D) Representative dot-plot graphs (annexin V: green FL1 channel, x-axis; PI: red FL3 channel, y-axis) from three independent experiments showing the reduced apoptosis after bortezomib treatment (48 h) in SeAx cells overexpressing miR-125b-5p compared to the scrambled transfected ones. All quantitative data are shown as mean ± SD (*P< 0.05).

Subsequently, we studied the effect of miR-125b-5p on the CTCL cell sensitivity to bortezomib. Bortezomib downregulated cMyc (Figure 2A) and upregulated miR-125b-5p (Figure 2B) in SeAx and MyLa cells. SeAx and MyLa cells overexpressing miR-125b-5p exhibited a significantly reduced sensitivity to bortezomib (Figure 2C, D), whereas miR-125b-5p knockdown resulted in an increased apoptosis after the treatment (Figure S1C). We have therefore shown that miR-125b-5p is a modulator of cell sensitivity to bortezomib.

### Reciprocal Negative Regulatory Loop between cMyc and miR-125b-5p *in vitro*

**Figure 3:**
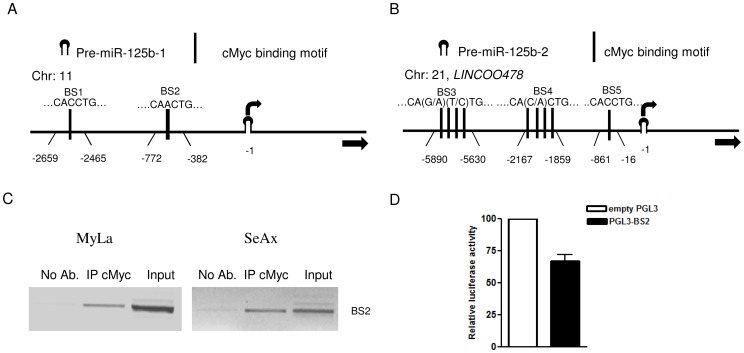
cMyc is a transcriptional repressor of miR-125b-5p. (A, B) Schematic representation of the predicted cMyc binding motifs (black segments) in the region 10 kb upstream of the pre-miR-125b-1 (A) and pre-miR-125b-2 (B) 5′ terminus (indicated as −1). The numbers indicate the amplicon positions relative to the 5′ terminus of pre-miR-125b-1 and pre-miR-125b-2. (C) ChIP assay on the BS2 fragment. Cross-linked chromatin was precipitated from SeAx and MyLa cells using cMyc antibody. (D) Luciferase activity (measured 48h after transfection) of the pGL3-promoter reporter construct containing the BS2 fragment compared to the empty construct in Hek-293 cells. The relative Firefly luciferase activity is showed after normalization to internal control Renilla luciferase and pGL3-promoter empty vector. Data are presented as mean ± SD.

We were then interested in the mechanism of miR-125b-5p repression. The miR-125b-5p mature form derives from two precursors, hsa-miR-125b-1 (chromosome 11) and hsa-miR-125b-2 (chromosome 21). By computer-based analyses using TESS-Transcription Element Search System [24] (http://www.cbil.upenn.edu/cgi-bin/tess/tess) we identified several putative cMyc binding sites in the region 10 kb upstream of the pre-miR-125b-1 and pre-miR-125b-2 5′ terminus. Two cMyc binding sites were predicted upstream to pre-miR-125b-1 and PCR amplicons for ChIP were designed covering the region between −2465 and −2659 (BS1), −382 and −772 (BS2) (Figure 3A). In the case of the pre-miR-125b-2, we designed three PCR amplicons ranging over the nine predicted cMyc binding regions; the first amplicon located at 16–861 bp (BS3), the second 1859–2167 bp (BS4), the third one (BS5) 5630–5890 bp upstream to the 5′ pre-miR-125b-2 terminus (Figure 3B). A significant enrichment of amplified DNA precipitated by the cMyc antibody was detected for BS2 (Figure 3C). No specific cMyc binding was found at the other putative binding sites. We then cloned the region containing the BS2 cMyc binding site into the pGL3-promotor reporter (Promega WI, USA) construct for a luciferase assay. cMyc overexpression led to a 40% decrease in the luciferase activity for the reporter construct containing the BS2 site (Figure 3D), while no changes were detected for the empty pGL3-promotor reporter. These results indicate that cMyc functions as a transcriptional repressor of miR-125b-5p.

**Figure 4:**
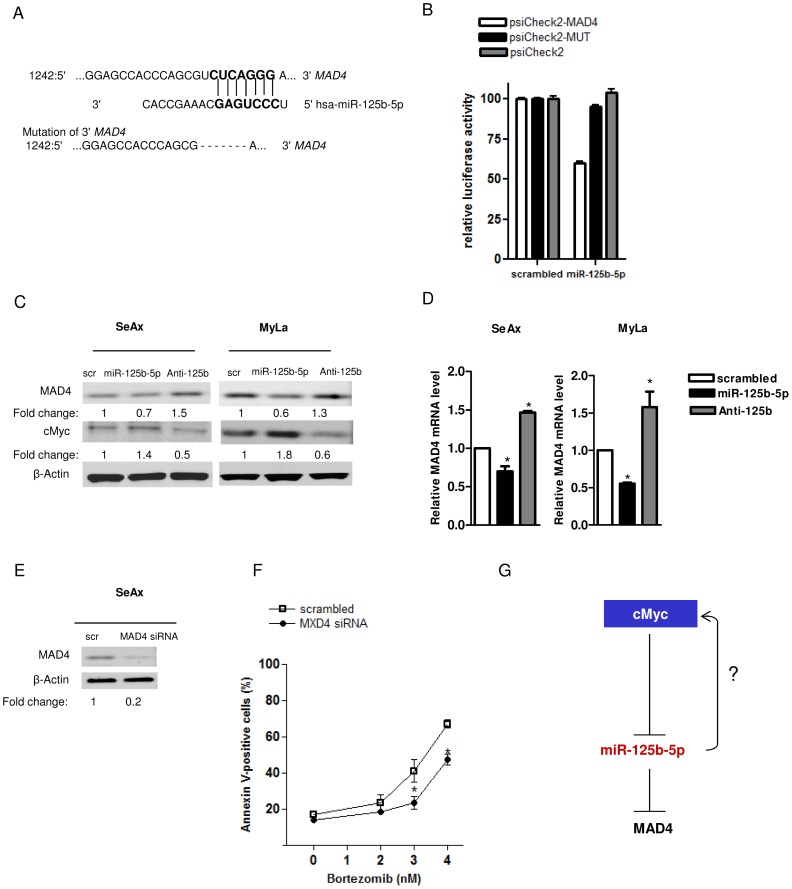
MAD4 is a direct target of miR-125b-5p. (A) The miR-125b-5p responsive element in *MAD4* gene 3′UTR and the base-paired to miR-125b-5p are showed on the top row of the schematic diagram. The psiCheck2 reporter constructs containing the *MAD4* 3′UTR (top row) or the mutated one (obtained by deleting the seed sequence of miR-125b-5p as shown in the lower row of the schematic diagram) were assayed. (B) Luciferase reporter assay (taken after 24h transfection in Hek-293 cells) showing that miR-125b-5p represses the luciferase activity of the reporter construct containing the MAD4 responsive element (psiCheck2-MAD4). Deletion of the miR-125b-5p responsive element in the MAD4 3′UTR (psiCheck2-Mut) rescued the luciferase activity. Renilla luciferase activities were normalized to the values of the internal Firefly and to the scrambled transfection control. (C) Detection of the MAD4 and cMyc protein levels in CTCL cells (SeAx and MyLa) after miR-125b-5p overexpression or downregulation. β-actin was the loading control and protein changes are showed as in Figure 1A. (D) Changes in the relative MAD4 mRNA level detected by qRT-PCR in CTCL cells. (E) Evaluation of the efficient MAD4 silencing in SeAx cells by Western Blot. (F) Decrease of the percentage of the annexin-V positive cells after MAD4 silencing in SeAx cells treated with bortezomib (48h) comparing to scrambled siRNA. (G) A mechanistic model describing the regulative loop involving cMyc and miR-125b-5p in cell resistance to bortezomib. cMyc induces the transcriptional inhibition of miR-125b-5p that directly inhibits MAD4 and induces cMyc accumulation. All quantitative data are shown as mean ± SD (*P< 0.05).

Our search to identify possible miR-125b-5p targets was performed using the online tools TargetScan [25] and Pictar [26] and predicted the 3′ UTR of the negative regulator of cMyc, *MAD4*, to contain one evolutionarily conservative seed sequence for miR-125b-5p (Figure 4A). Luciferase reporter assay revealed a direct inhibition of MAD4 by miR-125b-5p. miR-125b-5p significantly repressed the luciferase activity compared to scrambled oligonucleutide, while site directed mutagenesis of the complementary seed region counteracted the target gene repression (Figure 4B). In SeAx and MyLa cells ectopic expression or knockdown of miR-125b-5p resulted in respectively, a decrease and an upregulation of MAD4 both at the protein (Figure 4C) and mRNA level (Figure 4D). Accumulation of MAD4 protein level was also observed in cMyc overexpressing cells (Figure S1A). miR-125b-5p overexpression led to cMyc accumulation, suggesting that miR-125b-5p could modulate cMyc through a regulative loop. We next investigated whether the repression of MAD4 was functional in CTCL. Silencing of MAD4 did not affect cMyc protein and mRNA levels (Figure S2A, B), but it recapitulated the inhibiting effect of miR-125b-5p on the apoptosis induced by bortezomib (Figure 4E, F). All considered, our results reveal a role of miR-125b-5p in cell resistance to bortezomib and identify an auto regulatory circuit, on which cMyc regulates the expression of miR-125b-5p that, in turn, controls cMyc accumulation (Figure 4G).

### Inverse Correlation between cMyc and miR-125b-5p in Mycosis Fungoides

**Figure 5:**
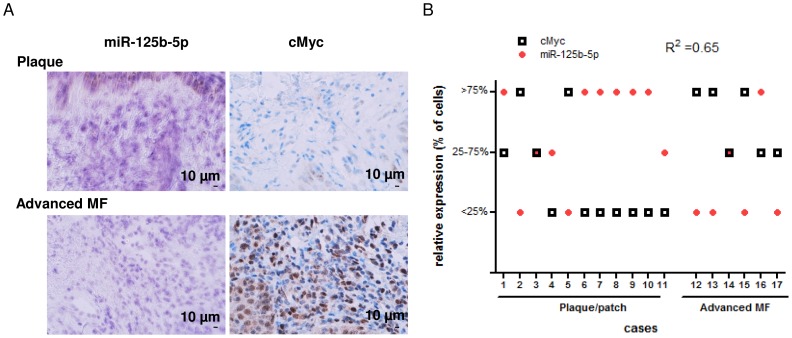
miR-125b-5p inversely correlates with cMyc expression in mycosis fungoides. (A) Representative cases from the 17 mycosis fungoides specimens (plaque (T2), n = 11; advanced mycosis fungoides (T3), n = 6) analyzed by IHC for cMyc and by ISH for detection of miR-125b-5p. Scale bar, 10 µm. (B) The 17 mycosis fungoides specimens were classified into three categories depending on the percentages of miR-125b-5p or cMyc positive tumour cells. The Pearson coefficient (R^2^ = 0.65; *P*< 0.05) indicates an inverse correlation between miR-125b-5p and cMyc expressions.

To investigate whether the reciprocal regulation of cMyc and miR-125b-5p is also seen *in vivo* in CTCL we analyzed 17 skin lesions from patients with mycosis fungoides (T2, n = 11; T3, n = 6) for cMyc and miR-125b-5p by immunohistochemistry (IHC) and *in situ* hybridization (ISH), respectively. In earlier stages (T2, plaques) cMyc expression was low in 72% cases (8/11), whereas high cMyc signalling was detected in the advanced mycosis fungoides (T3, tumors) (Figure 5A, B). Importantly, cMyc inversely correlated with miR-125b-5p expression in lesions of mycosis fungoides (Pearson coefficient R^2^ = 0.65; *P*<0.05; Figure 5B).

### miR-125b-5p Increases the Growth and Aggressiveness of CTCL *in vivo*

**Figure 6:**
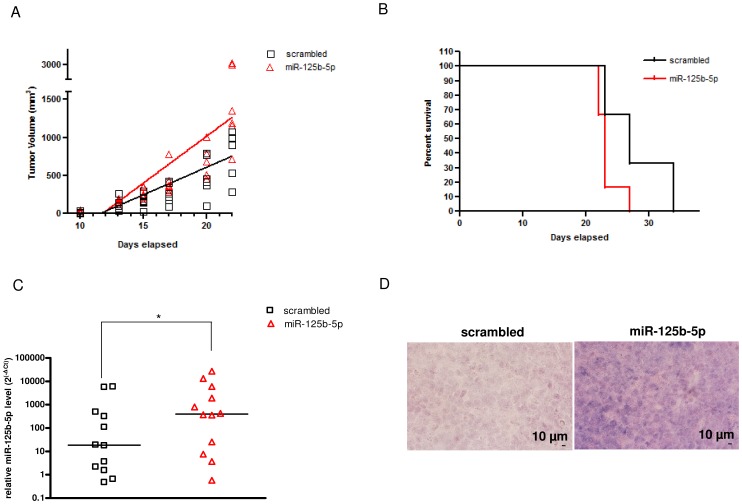
miR-125b-5p facilitates tumorigenesis *in vivo*. (A) Linear regression analysis of the *in vivo* tumor volume in 12 NSG mice (n = 6/group) after xenotransplantation of MyLa cells transfected with miR-125b-5p or scrambled oligonucleutide. The tumor growth rate represented by the slope of the linear regression was statistically different in the miR-125b-5p group compared to the scrambled one (*P* = 0.03). Each point represents a single measurement. (B) Kaplan-Meier survival curve showing a significant (Log-Rank Mantel Cox test *P* = 0.04) shorter median survival in the miR-125b-5p group compared to the scrambled one. (C) miR-125b-5p expression in miR-125b-5p and scrambled tumors was quantified by qRT-PCR (*P< 0.05). Each point represents the miR-125b-5p level in one tumor tissue and the line corresponds to the median value of miR-125b-5p in the miR-125b-5p and scrambled tumors. (D) A representative ISH from 6 cases of paraffin sections of miR-125b-5p or scrambled tumors using miR-125b-5p-specific LNA probe. miR-125b-5p expression was high in all tumors within the miR-125b-5p group, while it was almost undetectable in the tumors within the control group. Images were acquired using a Nikon D60 digital single-lens reflex (D-SLR) camera. Original magnification x20 for both panels. Scale bar, 10 µm.

The tumour development in CTCL may be modelled in mice by xenotransplantation of MyLa cells into NSG immunodeficient mice [42]. We have investigated whether miR-125b-5p overexpression confers a predicted increased tumorigenicity in CTCL. Although the mice invariably developed the tumour with a short onset (10 days), the median tumour growth was enhanced in the miR-125b-5p group compared to the scrambled control (Figure 6A; *P* = 0.03). Accordingly, overexpression of miR-125b-5p resulted in a significant (*P* = 0.04) shorter median survival (23 days) compared to the scrambled group (27 days, Figure 6B). Importantly, the increased levels of miR-125b-5p expression were maintained in the xenograft for the entire period of the experiment (Figure 6C, D). We thus conclude that miR-125b-5p provides a significant survival advantage *in vivo* to CTCL cells.

## Discussion

This study elucidates a new mechanism of action of miR-125b-5p in cancer cells *via* direct targeting of the cMyc antagonist, MAD4. cMyc, in turn directly inhibits transcription of miR-125b-5p. The miR-125b-5p signal has an important functional significance in the regulation of lymphoma tumor development and resistance of lymphoma cells to chemotherapy. Bortezomib treatment induced miR-125b-5p that exerted a cytoprotective effect by reducing the drug-induced apoptosis. cMyc reversed the resistance to bortezomib through the transcriptional repression of miR-125b-5p.

miR-125b-5p has been previously shown to act as an oncomiR in leukemogenesis [43]. The major carcinogenic event is the translocation of the miR-125b locus into the immunoglobulin heavy chain enhancer in lymphoblastic leukemia (t(11;14)(q24;q32)) [44] and the miR-125b activation by the chromosomal translocation t(2;11)(p21;q23) in acute myeloid leukemia [45]. miR-125b-5p targets multiple genes involved in the regulation of apoptosis including *BAK1, BCL3, PUMA* and *STAT3*, but depending on the cell type, miR-125b-5p contributes either to oncogenesis or to tumor suppression [43]. For instance, overexpression of miR-125b-5p leads to apoptosis in multiple myeloma cells [46], but it provides a survival advantage in T-ALL [47], [48].

Our results showed that miR-125b-5p, by targeting MAD4, contributes to a new mechanism of action applicable to T-cell lymphoma. MAD protein family, including the closely related transcriptional repressors MAD1, MAD3, MAD4 and MXI1, antagonizes cMyc function by competing with cMyc for the binding to MAX [49]. MAD proteins were proposed to act as tumor suppressors in glioma [50] and medullablastoma [51]. The significance of MAD in leukemia and lymphoma is poorly understood, but it has been observed that mutations in MAD1 and MXI1 correlated with a poor clinical outcome in acute leukemia [52]. Our findings reveal a role of miR-125b-5p/MAD4 signaling axis in T-lymphomas. Upregulation of miR-125b-5p downregulated MAD4 and increased the tumorigenic potential of the CTCL line MyLa and the cell resistance to bortezomib.

The induction of miR-125b-5p also caused accumulation of cMyc protein. However, it seems that another mechanism, rather than the repression of MAD4, contributes to this effect since the silencing of MAD4 decreased the apoptosis rate induced by bortezomib. In the same time MAD4 failed to induce upregulation of cMyc, both at the protein and mRNA level. We postulate that the effect of MAD4 on apoptosis could be separated from the direct antagonism of cMyc. This data are consistent with previous studies [53], showing that cMyc and MAD might modulate the same set of genes involved in proliferation and transformation, as well as some different ones involved in the apoptotic response. The authors suggested that other cMyc functions, such as the interaction with the protein MIZ-1 [54], are not subject to the MAD antagonism and could contribute to the cMyc target specificity and transcriptional activity.

cMyc is a hallmark of aggressive, poorly differentiated tumors and it plays multiple roles in driving leukemogenesis, including the ability to elicit tumors in experimental animals and to promote proliferation and growth in a number of cell lines and primary cells derived from lymphoid and myeloid leukemia [55], [56]. In CTCL, cMyc accumulation was associated with poor clinical outcome of the advanced stages of mycosis fungoides and Sèzary syndrome [15], [16]. A new layer of complexity is highlighted here and we reported that cMyc sensitized CTCL cells to apoptosis, analogously to previous reports in multiple myeloma, melanoma, breast and cervical carcinoma [12]–[14], [57]. Understanding the mechanisms through which cMyc modulates apoptosis would lead to improve the strategies that therapeutically target cMyc. It has been proposed that cMyc could directly, or through the p53 tumor suppressor, activate the proapoptotic protein BAX and induce the release of cytochrome-c from the mitochondria [57]. Here we identified another possible mechanism linking cMyc to apoptosis, which involves the direct transcriptional repression of the oncomiR-125b-5p. Overexpression of cMyc induced downregulation of miR-125b-5p and led to augmented apoptosis due to the proteasome blockade. Silencing of cMyc was associated with a high miR-125b-5p expression level and drug resistance.

Concluding, we discovered a new mechanism involving cMyc, through which miR-125b-5p could affect the clinical outcome of proteasome inhibitors. Our work provides a rationale for combining bortezomib and miRNAs to sensitize therapy-resistant cancer to chemotherapy. This may be especially relevant for the tumor regression of low grade lymphomas that are resistant to bortezomib treatment as a single therapeutic agent.

## Supporting Information

Figure S1 **miR-125b-5p silencing increases cell sensitivity to bortezomib.** (A) cMyc and MXD4 protein levels were assessed by western blot analysis in SeAx cells transfected either with cMyc expression plasmid (cMyc) or control vector (Ctr). cMyc band intensities was normalized to β-actin values. (B) Increase of the percentage of apoptotic annexin-V positive cells after bortezomib treatment (48 h) in Seax cells transfected with cMyc expression plasmid (cMyc) compared to the ones transfected with the control vector (Ctr). (C) miR-125b-5p LNA inhibitor augments the apoptosis induced by bortezomib treatment (48h). Bar graph shows the percentage of annexin V-positive cells in the total cell population. Data are presented as mean ± SD (*P<0.05).
(TIF)

Figure S2 **MAD4 silencing is not affecting cMyc level.** (A–B) Efficient MAD4 silencing in SeAx cells was not affecting the cMyc protein level measured by western blot analysis (A) and cMyc mRNA expression detected by RT-PCR (B) at 24h transfection.
(TIF)

Table S1 **miRNAs differentially expressed in cMyc-knockdown MyLa cells.** miRNAs changes are represented as fold regulation. In the case of fold-change values greater than one, the fold-regulation is equal to the fold-change. For the fold-change values lower than one, the fold-regulation is the negative inverse of the fold-change.
(DOC)
