# Supplementary figures and images for: cMyc/miR-125b-5p Signalling Determines Sensitivity to Bortezomib in Preclinical Model of Cutaneous T-Cell Lymphomas
Source: PLoS One. 2013 Mar 19;8(3):e59390. doi: 10.1371/journal.pone.0059390 (PMC3602111; doi:10.1371/journal.pone.0059390)

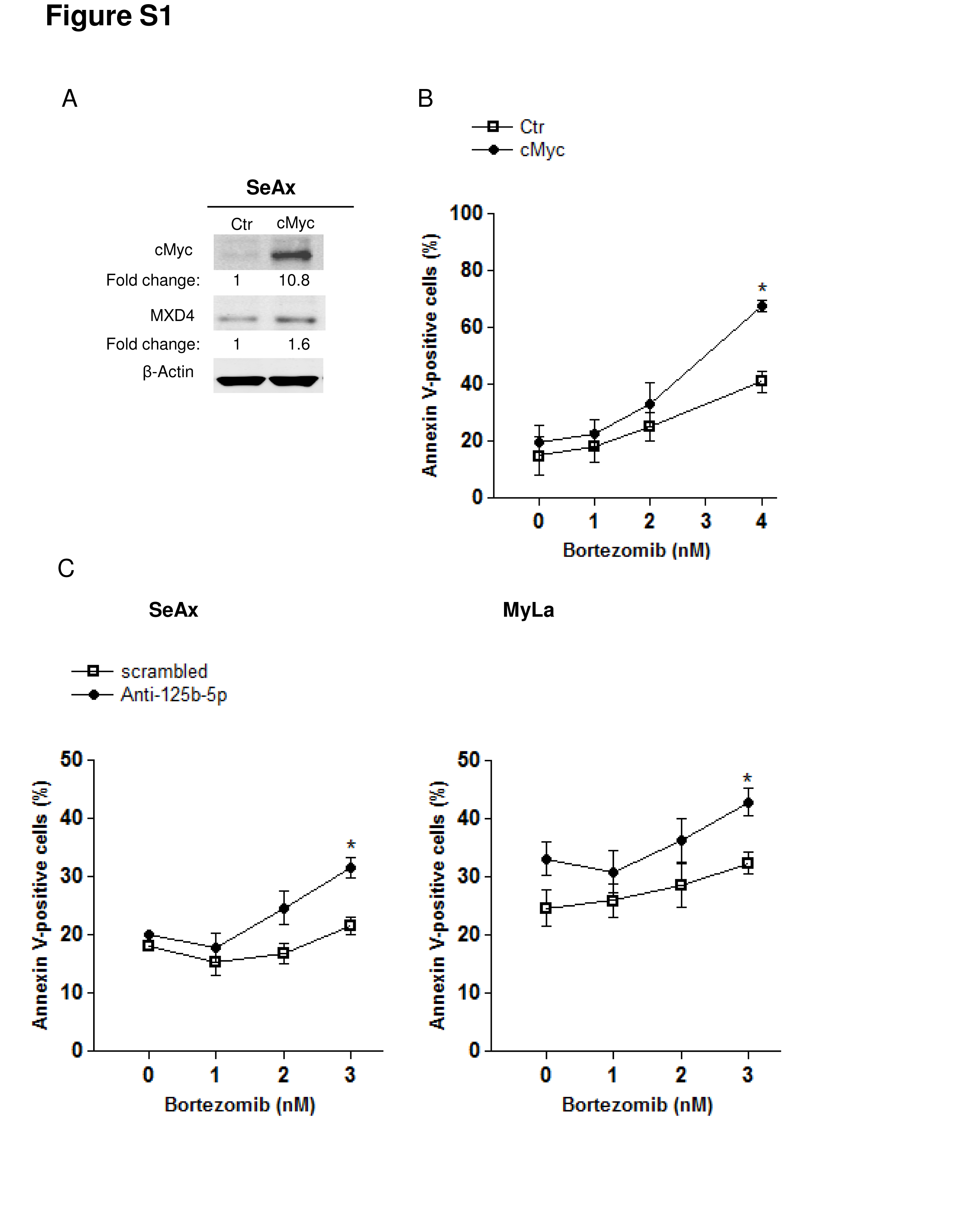

Supplement: Figure S1 — miR-125b-5p silencing increases cell sensitivity to bortezomib. (A) cMyc and MXD4 protein levels were assessed by western blot analysis in SeAx cells transfected either with cMyc expression plasmid (cMyc) or control vector (Ctr). cMyc band intensities was normalized to β-actin values. (B) Increase of the percentage of apoptotic annexin-V positive cells after bortezomib treatment (48 h) in Seax cells transfected with cMyc expression plasmid (cMyc) compared to the ones transfected with the control vector (Ctr). (C) miR-125b-5p LNA inhibitor augments the apoptosis induced by bortezomib treatment (48h). Bar graph shows the percentage of annexin V-positive cells in the total cell population. Data are presented as mean ± SD (*P<0.05). (TIF) [file pone.0059390.s001.tif]

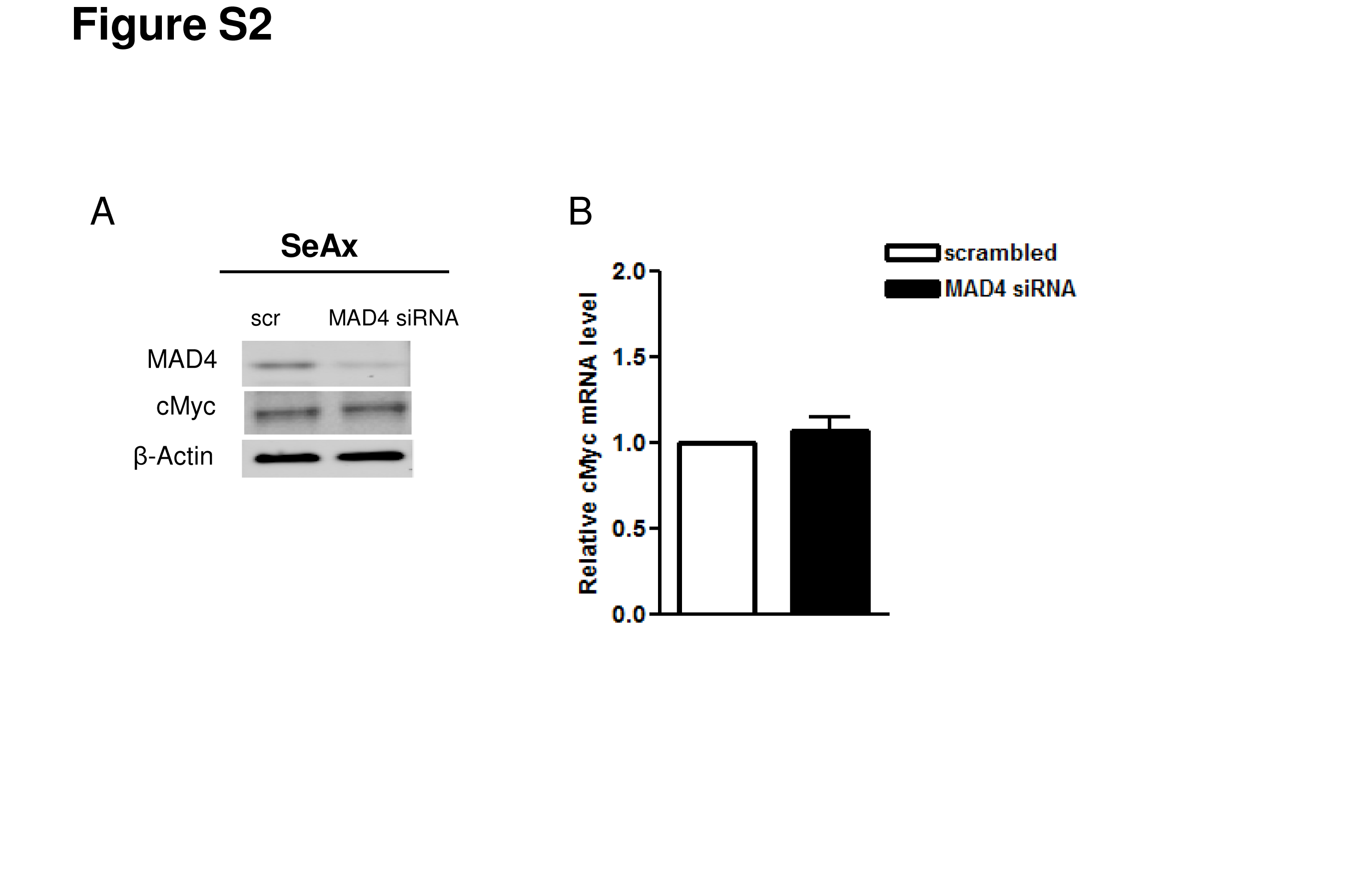

Supplement: Figure S2 — MAD4 silencing is not affecting cMyc level. (A–B) Efficient MAD4 silencing in SeAx cells was not affecting the cMyc protein level measured by western blot analysis (A) and cMyc mRNA expression detected by RT-PCR (B) at 24h transfection. (TIF) [file pone.0059390.s002.tif]
