# Supplementary material for: cMyc/miR-125b-5p Signalling Determines Sensitivity to Bortezomib in Preclinical Model of Cutaneous T-Cell Lymphomas
Source: PLoS One. 2013 Mar 19;8(3):e59390. doi: 10.1371/journal.pone.0059390 (PMC3602111; doi:10.1371/journal.pone.0059390)
Supplement: Table S1 — miRNAs differentially expressed in cMyc-knockdown MyLa cells. miRNAs changes are represented as fold regulation. In the case of fold-change values greater than one, the fold-regulation is equal to the fold-change. For the fold-change values lower than one, the fold-regulation is the negative inverse of the fold-change. (DOC) [file pone.0059390.s003.doc]

**Table S1.**

| **Detector** | **Fold**  **Regulation** | **Detector** | **Fold**  **Regulation** | **Detector** | **Fold**  **Regulation** | **Detector** | **Fold**  **Regulation** |
| --- | --- | --- | --- | --- | --- | --- | --- |
| hsa-miR-96-5p | 11,92 | hsa-miR-142-5p | -6,17 | hsa-miR-19b-2-5p | -4,48 | hsa-miR-4300 | -1452392,55 |
| hsa-miR-185-5p | 4,91 | hsa-miR-9-5p | -546,85 | hsa-miR-33b-5p | -9,40 | hsa-miR-517-5p | -1238360,56 |
| hsa-miR-200c-3p | 4,01 | hsa-miR-27b-3p | -5,64 | hsa-miR-374b-3p | -7,69 | hsa-miR-4265 | -668236,84 |
| hsa-miR-125b-5p | 376,11 | hsa-miR-32-5p | -5934,87 | hsa-miR-20a-3p | -5,88 | hsa-miR-3661 | -174,85 |
| hsa-miR-346 | 4.461,56 | hsa-miR-26b-5p | -550,65 | hsa-miR-221-5p | -5,64 | hsa-miR-2116-3p | -152,22 |
| hsa-miR-129-5p | 7,80 | hsa-let-7g-5p | -119,02 | hsa-miR-143-5p | -12474,26 | hsa-miR-921 | -109,14 |
| hsa-miR-513a-5p | 4.309,59 | hsa-miR-30d-5p | -4,33 | hsa-miR-205-3p | -445994,58 | hsa-miR-3176 | -4,72 |
| hsa-miR-548a-5p | 166289 | hsa-miR-130a-3p | -9,75 | hsa-miR-224-3p | -57715,32 | hsa-miR-3167 | -10,56 |
| hsa-miR-431-5p | 7,13 | hsa-miR-7-5p | -5,26 | hsa-miR-491-5p | -39148,43 | hsa-miR-3184-5p | -40063,48 |
| hsa-miR-298 | 12,32 | hsa-miR-100-5p | -124,07 | hsa-miR-548e | -2396,42 | hsa-miR-3065-5p | -9,99 |
| hsa-miR-146b-3p | 134,83 | hsa-miR-523-3p | -2471005,29 | hsa-miR-548l | -10,10 | hsa-miR-4290 | -6,41 |
| hsa-miR-1908 | 69,31 | hsa-miR-647 | -4,91 | hsa-miR-548-3p | -8,26 | hsa-miR-3161 | -398,93 |
| hsa-miR-191-3p | 103,61 | hsa-miR-542-3p | -18,61 | hsa-miR-573 | -34,46 | hsa-miR-877-3p | -4,86 |
| hsa-miR-297 | 12239150 | hsa-miR-579 | -7,83 | hsa-miR-593-3p | -54,07 | hsa-miR-101-5p | -4,23 |
| hsa-miR-299-5p | 73,26 | hsa-miR-595 | -101,71 | hsa-miR-1324 | -17,35 | hsa-miR-550a-3p | -6,02 |
| hsa-miR-302b-5p | 20,46 | hsa-miR-206 | -1654923,77 | hsa-miR-627 | -5621,22 | hsa-miR-1914-3p | -7,36 |
| hsa-miR-320b | 4,07 | hsa-miR-363-3p | -14148,22 | hsa-miR-218-1-3p | -62288,00 | hsa-miR-514b-5p | -37,53 |
| hsa-miR-185-3p | 48,28 | hsa-miR-148a-3p | -50,86 | hsa-miR-218-2-3p | -258,08 | hsa-miR-4309 | -68,12 |
| hsa-miR-223-5p | 4,40 | hsa-miR-650 | -219,54 | hsa-miR-23a-5p | -5,90 | hsa-miR-3156-5p | -8,46 |
| hsa-miR-26a-1-3p | 1174,91 | hsa-miR-484 | -4,50 | hsa-miR-449b-3p | -584,75 | hsa-miR-4263 | -6,41 |
| hsa-miR-34a-3p | 18929,30 | hsa-miR-549a | -55045,36 | hsa-miR-3152-3p | -1698,45 | hsa-miR-3170 | -8,06 |
| hsa-miR-374a-3p | 10,33 | hsa-miR-429 | -5,77 | hsa-miR-4264 | -77,17 |  |  |
| hsa-miR-3924 | 13777,25 | hsa-miR-589-5p | -10,61 | hsa-miR-3671 | -41668,42 |  |  |
| hsa-miR-3657 | 1978,24 | hsa-miR-345-5p | -5,05 | hsa-miR-466 | -45914,64 |  |  |
| hsa-miR-3941 | 59,71 | hsa-miR-17-3p | -8,73 | hsa-miR-3175 | -6,01 |  |  |
| hsa-miR-589-3p | 7,67 | hsa-miR-455-3p | -144,34 | hsa-miR-3681-3p | -5,05 |  |  |
| hsa-miR-4312 | 22278,02 | hsa-let-7f-2-3p | -5,25 | hsa-miR-550a-5p | -83,87 |  |  |
| hsa-miR-4261 | 20,16 | hsa-let-7i-3p | -4,87 | hsa-miR-4310 | -1472667,19 |  |  |
| hsa-miR-3147 | 180,18 | hsa-miR-106b-3p | -7,64 | hsa-miR-598 | -1763487,60 |  |  |
| hsa-miR-1911-5p | 254,23 | hsa-miR-126-5p | -12,24 | hsa-miR-3914 | -1679965,30 |  |  |
| hsa-miR-19a-5p | -5,83 | hsa-miR-525-5p | -1482910,40 | hsa-miR-759 | -186,11 |  |  |
